# Correlation Analysis between Gut Microbiota Alterations and the Cytokine Response in Patients with Coronavirus Disease during Hospitalization

**DOI:** 10.1128/spectrum.01689-21

**Published:** 2022-03-07

**Authors:** Taketoshi Mizutani, Aya Ishizaka, Michiko Koga, Kazuhiko Ikeuchi, Makoto Saito, Eisuke Adachi, Seiya Yamayoshi, Kiyoko Iwatsuki-Horimoto, Atsuhiro Yasuhara, Hiroshi Kiyono, Tetsuro Matano, Yutaka Suzuki, Takeya Tsutsumi, Yoshihiro Kawaoka, Hiroshi Yotsuyanagi

**Affiliations:** a Division of Infectious Diseases, Advanced Clinical Research Center, the Institute of Medical Science, The University of Tokyo, Tokyo, Japan; b Department of Computational Biology and Medical Sciences, Graduate School of Frontier Sciences, The University of Tokyo, Chiba, Japan; c Department of Infectious Diseases and Applied Immunology, IMSUT Hospital of Institute of Medical Science, the University of Tokyo, Tokyo, Japan; d Division of Virology, Department of Microbiology and Immunology, Institute of Medical Science, The University of Tokyo, Japan; e Research Center for Global Viral Infections, National Center for Global Health and Medicine, Tokyo, Japan; f International Research and Development Center for Mucosal Vaccines, the Institute of Medical Science, The University of Tokyo, Tokyo, Japan; g CU-UCSD Center for Mucosal Immunology, Allergy and Vaccines (cMAV), Department of Medicine, University of California San Diego, San Diego, California, USA; h Future Medicine Education and Research Organization, Chiba University, Chiba, Japan; i AIDS Research Center, National Institute of Infectious Diseases, Tokyo, Japan; j Department of AIDS Vaccine Development, IMSUT Hospital, the Institute of Medical Science, The University of Tokyo, Tokyo, Japan; University of Georgia

**Keywords:** COVID-19 pathogenesis, gut microbiota, inflammation, immune response, microbiome

## Abstract

The role of the intestinal microbiota in coronavirus disease 2019 (COVID-19) is being elucidated. Here, we analyzed the temporal changes in microbiota composition and the correlation between inflammation biomarkers/cytokines and microbiota in hospitalized COVID-19 patients. We obtained stool specimens, blood samples, and patient records from 22 hospitalized COVID-19 patients and performed 16S rRNA metagenomic analysis of stool samples over the course of disease onset compared to 40 healthy individual stool samples. We analyzed the correlation between the changes in the gut microbiota and plasma proinflammatory cytokine levels. Immediately after admission, differences in the gut microbiota were observed between COVID-19 patients and healthy subjects, mainly including enrichment of the classes Bacilli and Coriobacteriia and decrease in abundance of the class Clostridia. The bacterial profile continued to change throughout the hospitalization, with a decrease in short-chain fatty acid-producing bacteria including *Faecalibacterium* and an increase in the facultatively anaerobic bacteria Escherichia*-Shigella*. A consistent increase in *Eggerthella* belonging to the class Coriobacteriia was observed. The abundance of the class Clostridia was inversely correlated with interferon-γ level and that of the phylum Actinobacteria, which was enriched in COVID-19, and was positively correlated with gp130/sIL-6Rb levels. Dysbiosis was continued even after 21 days from onset. The intestines tended to be an aerobic environment in hospitalized COVID-19 patients. Because the composition of the gut microbiota correlates with the levels of proinflammatory cytokines, this finding emphasizes the need to understand how pathology is related to the temporal changes in the specific gut microbiota observed in COVID-19 patients.

**IMPORTANCE** There is growing evidence that the commensal microbiota of the gastrointestinal and respiratory tracts regulates local and systemic inflammation (gut-lung axis). COVID-19 is primarily a respiratory disease, but the involvement of microbiota changes in the pathogenesis of this disease remains unclear. The composition of the gut microbiota of patients with COVID-19 changed over time during hospitalization, and the intestines tended to be an aerobic environment in hospitalized COVID-19 patients. These changes in gut microbiota may induce increased intestinal permeability, called leaky gut, allowing bacteria and toxins to enter the circulatory system and further aggravate the systemic inflammatory response. Since gut microbiota composition correlates with levels of proinflammatory cytokines, this finding highlights the need to understand how pathology relates to the gut environment, including the temporal changes in specific gut microbiota observed in COVID-19 patients.

## INTRODUCTION

Severe acute respiratory syndrome coronavirus 2 (SARS-CoV-2) causes respiratory infection and lung damage, and it multiplies in and infects human small intestinal cells and duodenum (1, 2). SARS-CoV-2 RNA has been detected in feces, suggesting gastrointestinal tract involvement in viral infection (3, 4). The intestinal epithelium is densely populated with various microorganisms, and the intestinal microbiota has been reported to play an important role in maintaining immune homeostasis (5, 6). Coronavirus disease (COVID-19) mainly manifests with respiratory symptoms, but many cases with gastrointestinal symptoms, such as diarrhea, have also been reported (7).

In clinical reports of SARS-CoV-1 published in the early 2000s, diarrhea was noted in approximately 20% of the patients (8). During the current COVID-19 pandemic, Zuo et al. reported changes in the intestinal microbiota of hospitalized patients, and Yeoh et al. found that the abundance of several gut commensals known to have immunomodulatory effects, such as Faecalibacterium prausnitzii, Eubacterium rectale, and bifidobacteria, was decreased in patients with COVID-19 and that dysbiosis continued after recovery (9, 10). A recent study that followed up with 30 patients with COVID-19 reported that the gut microbiota richness did not recover, even after 6 months (11). Furthermore, elevated inflammatory cytokine and blood biomarker levels, and the gut bacterial composition, were stratified according to disease severity (9).

A limited amount of research has been performed on COVID-19 and the gut microbiota, but a growing number of studies have suggested that, in COVID-19 patients, gut microbiota alterations have some immune activation-related effect on the pathogenesis (12). A major challenge for research on gut microbial changes associated with COVID-19 is to overcome practical difficulty in collecting research specimens with considering a broad population stratified by region, ethnicity, gender, and age under the current overwhelmed clinical settings.

As one approach to understanding the pathogenesis of COVID-19, it is important to accumulate information on how the intestinal environment, including the microbiota, is changed by viral infection. In addition, because COVID-19 is characterized by a rapid change in pathology from minor symptoms resembling the common cold to severe pneumonia, we hypothesized that the intestinal microbiota may change as the disease progresses. Therefore, we analyzed the correlation between immune activation and temporal changes in the intestinal microbiota caused by SARS-CoV-2 infection in hospitalized COVID-19 patients, with the aim of expanding the information and enriching the evidence on the interaction between COVID-19 and the intestinal tract.

## RESULTS

### COVID-19 patient cohort and healthy subjects.

**FIG 1:**
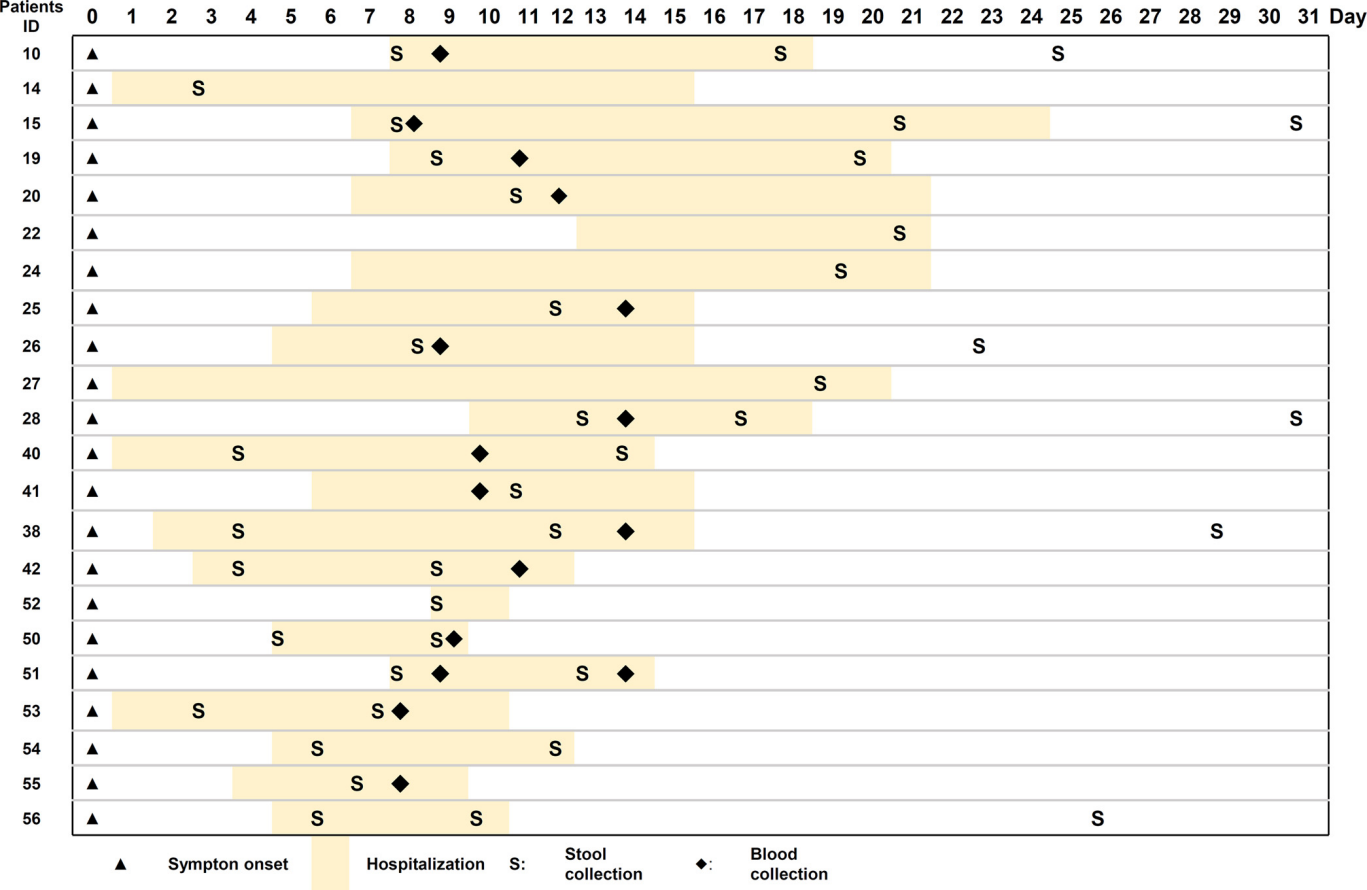
Schematic representation of the time course of disease onset and hospitalization duration of COVID-19 patients. Day 0 represents the day of disease onset. The date of stool and blood specimen collection is indicated.

Blood and stool samples were collected between February and August 2020 from 22 patients who had COVID-19 that had been confirmed by SARS-CoV-2 RT-qPCR and had been admitted to the IMSUT hospital. The demographic and clinical characteristics of these patients are shown in Table 1. All the patients enrolled in this study were infected with the original SARS-COV-2 strains (Wuhan and Wuhan/D614G strains). A non-COVID-19 control cohort of 40 healthy adults was recruited at the University of Tokyo in 2017 before the COVID-19 pandemic (13). The non-COVID-19 cohort consisted of 40 male individuals with a median age of 42 years (range, 23–60 years). The COVID-19 cohort comprising 3 female and 19 male patients with COVID-19; the median age was 42 years (range, 18–67 years). As per the severity classification, 7 patients had mild disease (30%), 12 had moderate disease (57%), and 3 patients had severe disease (13%), but no patients needed the forced ventilation. Of the 22 patients with COVID-19, 12 had comorbidities, including diabetes (one patient), pulmonary emphysema (two patients), dyslipidemia (two patients), bronchial asthma (three patients), and hypertension (three patients). Eight had received antibiotics at admission (Table S1 in the supplemental material). All patients recovered and were discharged after a mean hospital stay of 10.2 days (range, 2–20 days) (Fig. 1). Table S1 summarizes the treatments prescribed to each patient during their hospitalization. The current study was conducted before steroids were recommended for severe COVID-19 patients. No patients received steroids.

**TABLE 1:** Characteristics of COVID-19 patients

Description	Cases (*n* = 22)
Age: median, (IQR; interquartile range)	42 (18–67)
Gender male: *n* (ratio %)	18 (81.8%)
BMI (body mass index: kg/m^2^ median, (IQR)	24.2 (20.3–40.0)
Antibiotics: *n* (ratio %)	8 (36.4%)
Probiotics: *n* (ratio %)	6 (27.3%)
Symptoms during hospitalization: *n* (ratio %)	
Fever (≥37.5°C)	9 (40.9%)
Respiratory symptoms: *n* (ratio %)	
Cough	14 (63.6%)
Sore throat	6 (27.3%)
Dyspnea	8 (36.4%)
Diarrhea: *n* (ratio %)	6 (27.3%)
Chest CT (computed tomography) image: *n* (ratio %)	
Pneumonia	15 (68.2%)
Severity	
Mild	7 (31.8%)
Moderate	12 (54.5%)
Severe	3 (13.6%)

### Diversity analysis of gut microbiota in patients with COVID-19.

**FIG 2:**
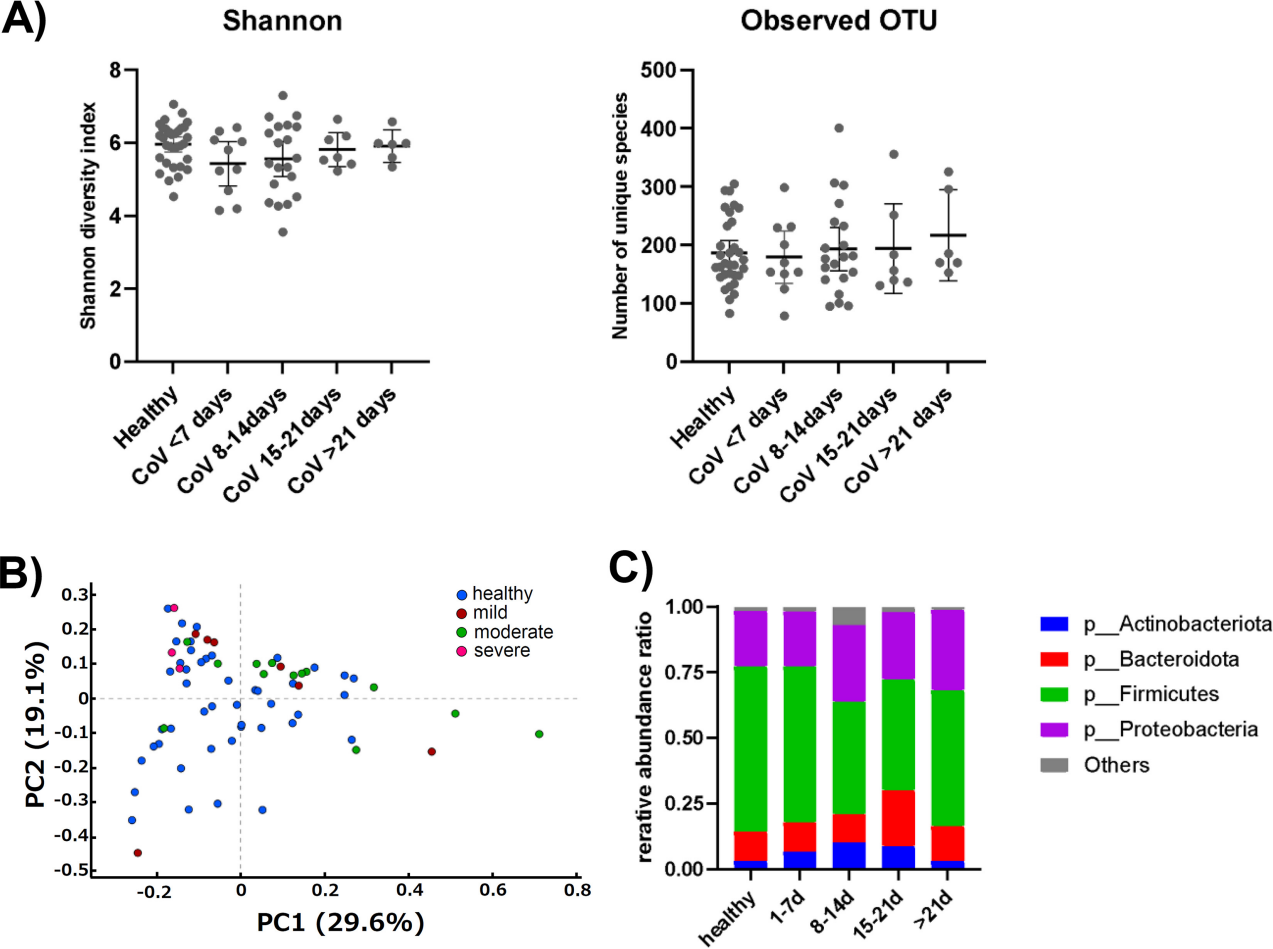
Differences in the composition of gut microbiota between healthy subjects and COVID-19 patients. (A) Diversity analysis of gut microbiota in COVID-19 patients. Shannon analysis (left) and observed operational taxonomic units (OTUs) (right). Patients were classified in 7-day categories from onset. (B) Principal coordinate analysis between healthy subjects and COVID-19 patients by disease severity. (C) Average relative abundance at the phylum level of intestinal microbiota in COVID-19 patients.

SARS-CoV-2 viral RNA has been reported in the intestinal tract (3, 4) and infects intestinal cells and the duodenum, (1, 2), suggesting that it affects the intestinal microbiota composition. The stool samples were subjected to 16S rRNA sequencing analysis. Firstly, to investigate the changes in gut microbiota from the onset of COVID-19 to recovery, stool samples obtained from the patients were classified into four groups according to the date of collection: within 7 days of onset (10 samples), 8–14 days (17 samples), 15–21 days (7 samples), and after 21 days (6 samples). The alpha diversity of the four groups and healthy control was then analyzed; there was no significant difference in the Shannon diversity index analysis and the observed operational taxonomic units between them (Fig. 2A). Next, we performed principal coordinate analysis of the gut microbiota of the 22 patients according to disease severity (Fig. 2B). For all severities (mild, moderate, and severe), the composition of the gut microbiota of the COVID-19 cohort overlapped with that of the healthy cohort, and no clear clustering differences were observed. However, there are some samples in the moderate and mild categories that are further distances than the rest of the samples. Thus, the α-diversity index did not differ throughout the treatment period between the 22 COVID-19 affected subjects and the 40 healthy subjects, and no clear differences were observed in β-diversity comparing patients with different severity of disease and healthy subjects.

### Changes in intestinal microbiota at the phylum level during the first 2 weeks of COVID-19.

We next analyzed when the change of the intestinal microbiota in patients with COVID-19 was observed after infection in the relative abundance at the phylum level (Fig. 2C). We categorized stool samples collected every week from the day of disease onset, assuming that the gut microbiota profile would change over time. We observed that the intestinal microbiota gradually changes immediately after the onset of the disease, with largest changes observed at 8–14 days after symptom onset. Among the four main phyla, Firmicutes, Proteobacteria, Bacteroidetes, and Actinobacteria, the COVID-19 patients showed a gradual decrease in abundance of Firmicutes from disease onset (59.3%), with a peak decrease noted at 8–21 days (42.2%), which was followed by recovery. The ratio of Proteobacteria showed 9% increase from 1–7 days to 8–14 days (29%). Bacteroidetes showed no change in the early stages of disease onset, but enrichment was observed in patients at 15–21 days (21.4%) after disease onset (11.2%). For Actinobacteria, enrichment gradually progressed from 8 to 14 days (10.3%) after disease onset (6.8%). The differences in relative abundance of the intestinal microbiota between patients with COVID-19 and healthy subjects are shown in Table 2. Only intestinal bacteria with an average relative abundance of 1% or more in healthy subjects were extracted. The relative abundance of bacteria in the stool of COVID-19 patients showed an increase in genera such as *Bifidobacterium*, *Bacteroides*, *Parabacteroides*, and Escherichia-*Shigella* and a decrease in genera such as *Faecalibacterium*, *Subdoligranulum*, *Dorea*, and Enterobacter from onset to day 14, as compared with the relative abundance of bacteria in the stool of healthy subjects. These observations, however, were not statistically significant.

**TABLE 2:** Relative abundance of gut microbiota in healthy cohorts and COVID patients

			Days from onset			
Taxa^*a*^	Association cohort	Healthy	1–7	8–14	15–21	>21
p__Actinobacteriota|c__Actinobacteria|o__Bifidobacteriales|f__Bifidobacteriaceae|g__Bifidobacterium	COVID	2.85	3.48	5.95	6.73	1.60
p__Bacteroidota|c__Bacteroidia|o__Bacteroidales|f__Bacteroidaceae|g__Bacteroides	COVID	8.67	9.81	9.72	19.26	10.22
p__Bacteroidota|c__Bacteroidia|o__Bacteroidales|f__Tannerellaceae|g__Parabacteroides	COVID	1.32	2.37	1.80	1.72	2.28
p__Firmicutes|c__Clostridia|o__Clostridia_UCG-014|f__Clostridia_UCG-014|g__Clostridia_UCG-014	Healthy	1.64	0.16	0.04	0.00	0.07
p__Firmicutes|c__Clostridia|o__Lachnospirales|f__Lachnospiraceae|__	Healthy	1.65	0.43	1.75	1.85	1.59
p__Firmicutes|c__Clostridia|o__Lachnospirales|f__Lachnospiraceae|g__Agathobacter	Healthy	2.48	0.18	1.15	0.64	0.10
p__Firmicutes|c__Clostridia|o__Lachnospirales|f__Lachnospiraceae|g__Blautia	COVID	4.34	6.60	3.86	8.40	1.50
p__Firmicutes|c__Clostridia|o__Lachnospirales|f__Lachnospiraceae|g__Dorea	Healthy	3.47	0.49	1.41	0.24	0.05
p__Firmicutes|c__Clostridia|o__Monoglobales|f__Monoglobaceae|g__Monoglobus	Healthy	1.23	0.48	0.39	0.45	1.87
p__Firmicutes|c__Clostridia|o__Oscillospirales|f__[Eubacterium]_coprostanoligenes_group|g__[Eubacterium]_coprostanoligenes_group	Healthy	1.71	0.12	1.62	0.90	3.29
p__Firmicutes|c__Clostridia|o__Oscillospirales|f__Ruminococcaceae|g__Faecalibacterium	Healthy	16.40	14.32	5.15	0.99	11.07
p__Firmicutes|c__Clostridia|o__Oscillospirales|f__Ruminococcaceae|g__Subdoligranulum	Healthy	10.75	7.08	6.30	6.17	9.34
p__Firmicutes|c__Negativicutes|o__Veillonellales-Selenomonadales|f__Selenomonadaceae|g__Megamonas	COVID	2.91	5.31	1.89	0.24	10.78
p__Fusobacteriota|c__Fusobacteriia|o__Fusobacteriales|f__Fusobacteriaceae|g__Fusobacterium	Healthy	1.18	0.89	1.08	0.68	0.03
p__Proteobacteria|c__Gammaproteobacteria|o__Enterobacterales|f__Enterobacteriaceae|__	Healthy	5.78	1.53	1.29	11.48	9.23
p__Proteobacteria|c__Gammaproteobacteria|o__Enterobacterales|f__Enterobacteriaceae|g__Enterobacter	Healthy	1.09	0.00	0.09	1.58	1.45
p__Proteobacteria|c__Gammaproteobacteria|o__Enterobacterales|f__Enterobacteriaceae|g__Escherichia-Shigella	COVID	12.50	20.98	30.66	11.44	17.82

a Only bacteria with a relative abundance of more than 1% of the intestinal bacteria in healthy individuals are described.

### Relative abundance of intestinal microbiota in COVID-19 patients and analysis of variation in intestinal microbiota over time after hospitalization.

**FIG 3:**
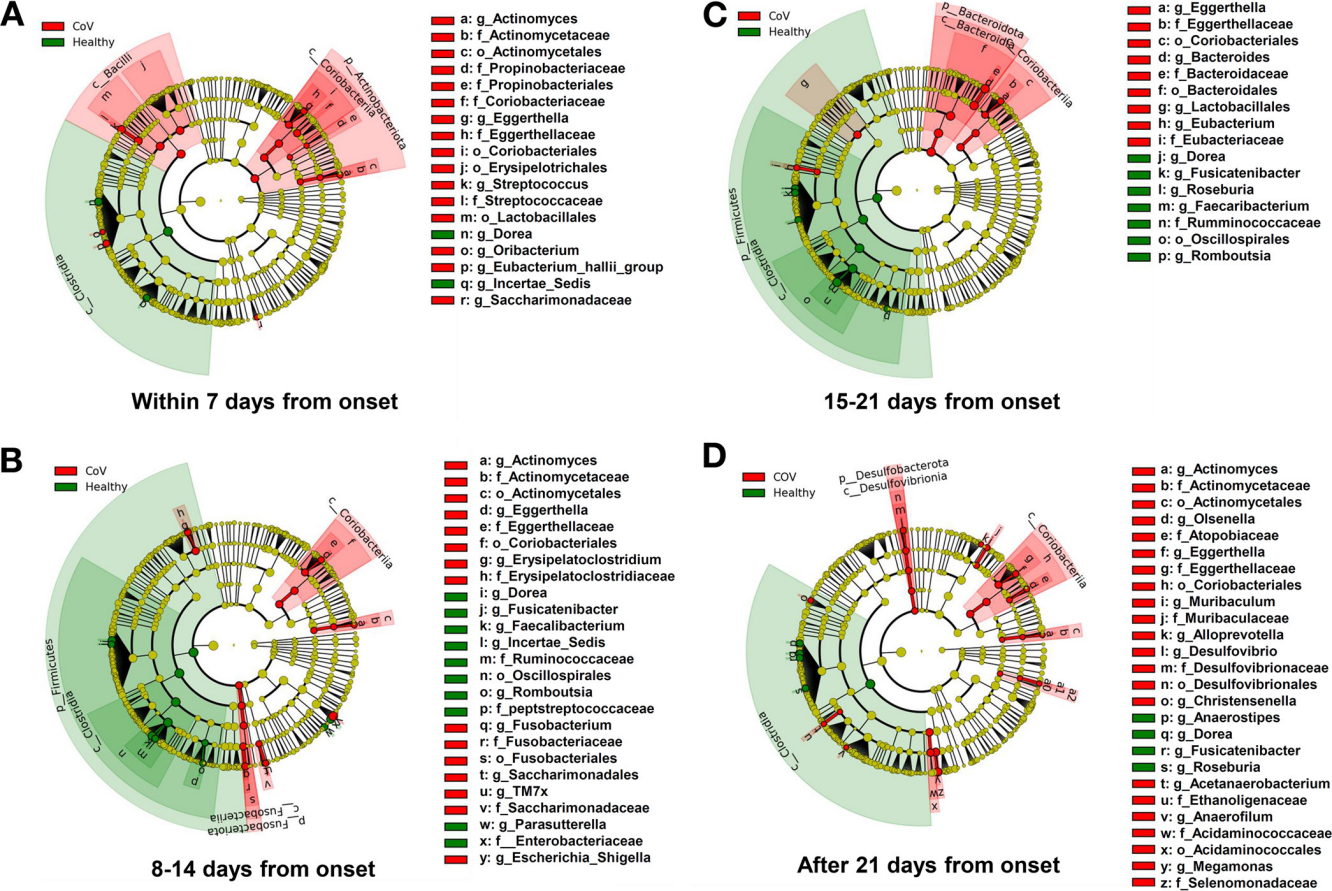
Comparative analysis of the intestinal microbiota of COVID-19 patients and healthy subjects. The stool samples of the patients were grouped chronologically from the onset of the disease by the date of collection (A, within 7 days from onset; B, 8–14 days from onset; C, 15–21 days from onset; and D, over 21 days). Changes in intestinal microbiota from hospitalization to postdischarge were analyzed using linear discriminant analysis effect size in comparison with healthy subjects.

For further in-depth study of the altered bacterial abundance, linear discriminant analysis (LDA) effect size (LEfSe) analysis was used to compare the changes in the intestinal microbiota of patients with COVID-19 and healthy subjects. First of all, consistently, a decrease in *Dorea* genus and an enrichment of *Eggerthella* genus and family Eggerthellaceae were observed in COVID-19 patients during hospitalization. Within 1 week of COVID-19 onset, the patients showed enrichment of the class Bacilli and the phylum Actinobacteria, including the class Coriobacteriia, whereas the class Clostridia showed slightly decreased abundance (*P* < 0.05; LEfSe; Fig. 3A; Table S2A).

At 8–14 days after onset, enrichment of the class Coriobacteriia was still observed. By contrast, a further decrease in the bacterial population was observed in the phylum Firmicutes, mainly in the class Clostridia (*P* < 0.05; LEfSe; Fig. 3B; Table S2B). This trend was also observed 15–21 days after disease onset, when enrichment of the phylum Bacteroidetes, including the class Bacteroidia, was observed (*P* < 0.05; LEfSe; Fig. 3C; Table S2C). Specifically, in patients with COVID-19, abundances of the order Oscillospirales; the family Ruminococcaceae; and genera *Fusicatenibuacterium*, *Faecalibacterium*, and *Romboutsia* were decreased within 15–21 days from disease onset. Of these, Ruminococcaceae contain butyrate-producing bacteria, and Faecalibacterium, which is classified as Ruminococcaceae, is a butyrate-producing bacterium (14). Furthermore, the abundance of the Escherichia*-Shigella* genus, a facultative anaerobic bacterium, was increased within 8–14 days from disease onset. After 21 days, the presence of class Clostridia remained reduced compared with that in healthy subjects (*P* < 0.05; LEfSe; Fig. 3D; Table S2D). Thus, the intestinal microbiota of patients with COVID-19 changed continuously over time, and these changes continued even after discharge.

Next, patients were classified according to the severity of illness (mild versus moderate/severe) and presence or absence of pneumonia by LEfSe analysis as well, but there was no significant difference in gut microbiota between patients during the 2 weeks from onset (data not shown). Of the 22 patients, 8 were also using antibiotics at the time of admission. Because antibiotic use is known to alter the profile of the gut microbiota (15), we examined this effect. Within 7 days from the onset of the disease, a difference was observed between antibiotic users and nonusers for sporadic bacteria belonging to phylum Firmicutes (Fig. S1A). This trend continued in the second week, with only minor antibiotic-induced changes in the gut microbiota among patients (Fig. S1B).

### Comparison of plasma cytokine levels and blood inflammation biomarkers in patients with COVID-19.

**FIG 4:**
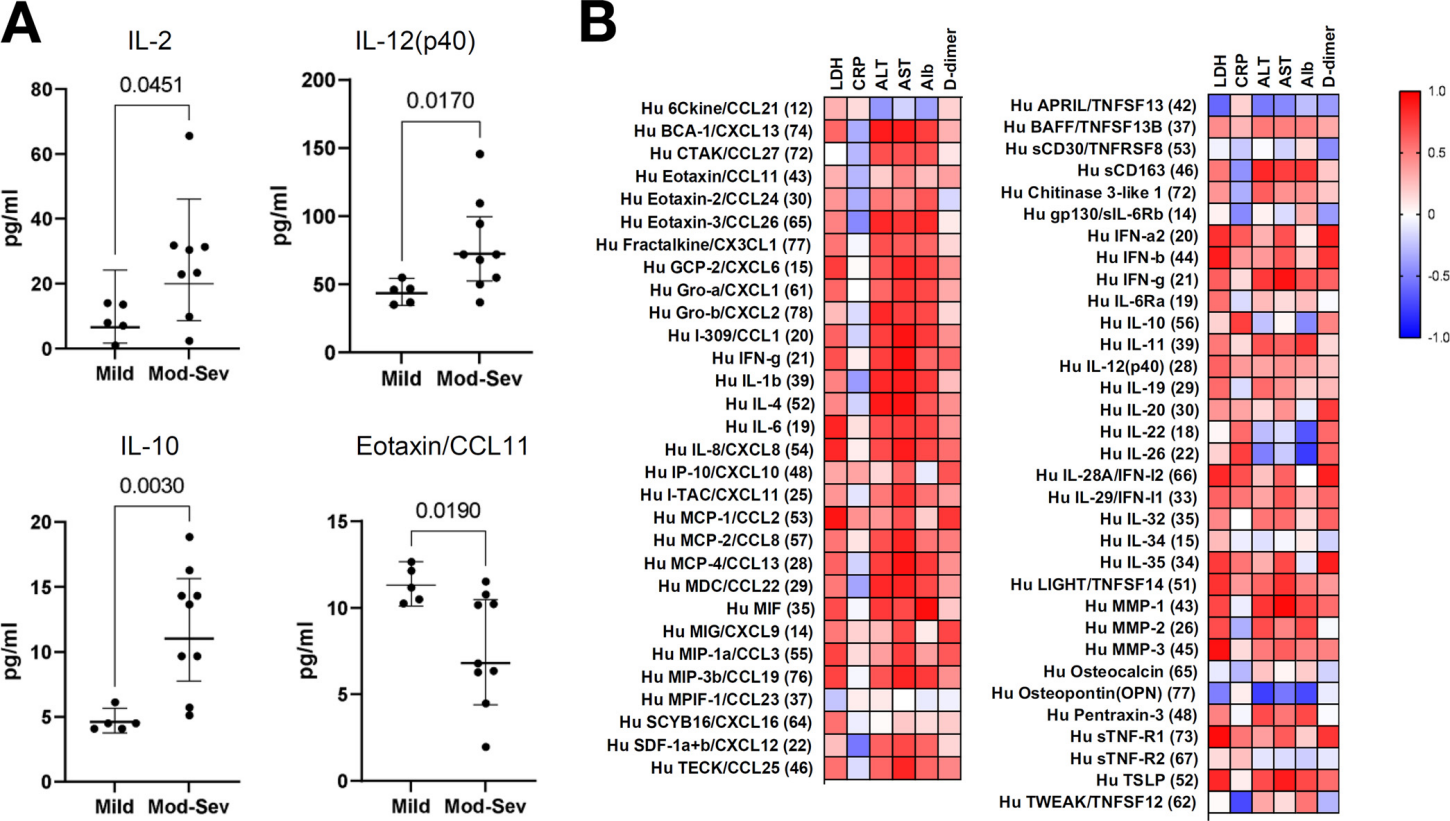
Correlation analysis between COVID-19 patients and plasma proinflammatory cytokine, chemokine, and biomarker levels in blood. (A) Comparative analysis between severity of illness and plasma inflammatory cytokine and chemokine levels using only patients during the first 7–14 days of illness. (B) Heatmap of the Spearman’s correlation analysis of plasma inflammatory cytokine and chemokine levels with blood biomarker levels in patients 7–14 days after onset. R values are represented as indicated by the color key.

Clinical studies on COVID-19 have shown that the viral infection activates T lymphocytes and mononuclear macrophages, which express many cytokines and cause severe inflammatory reactions in the lungs and other organs (16). Therefore, we compared the cytokine levels in the plasma of hospitalized patients according to disease severity. For cytokine analysis, only samples collected at 7–14 days from onset were evaluated to minimize the effect of onset date. The 16 affected patients whose samples corresponded to those collected during this period were classified into three groups according to the CDC classification: mild, moderate, and severe. Seven patients had mild disease, eight patients had moderate disease, and one patient had severe disease. We then divided the patients into two groups, patients with mild disease and those with moderate/severe disease whose computed tomography images showed pneumonia, and we compared the plasma inflammatory marker levels. The time from disease onset to blood collection did not significantly differ between the groups (Mann–Whitney test, data not shown). Moderate and severe disease groups had higher interleukin (IL)-2, IL-10, and IL-12 (p40) levels and lower Eotaxin/CC motif chemokine ligand 11 levels than the mild disease group (*P* < 0.05; Mann–Whitney test; Fig. 4A). In addition, correlation analysis of plasma cytokine levels with inflammatory blood biomarkers showed that alanine transaminase, aspartate transaminase, d-dimer, and lactate dehydrogenase levels were positively correlated with most inflammatory cytokine and chemokine levels, whereas C-reactive protein levels were positively correlated with some cytokine levels (Fig. 4B and Table S3).

### Association between gut microbiota and plasma cytokine level alterations in patients with COVID-19.

**FIG 5:**
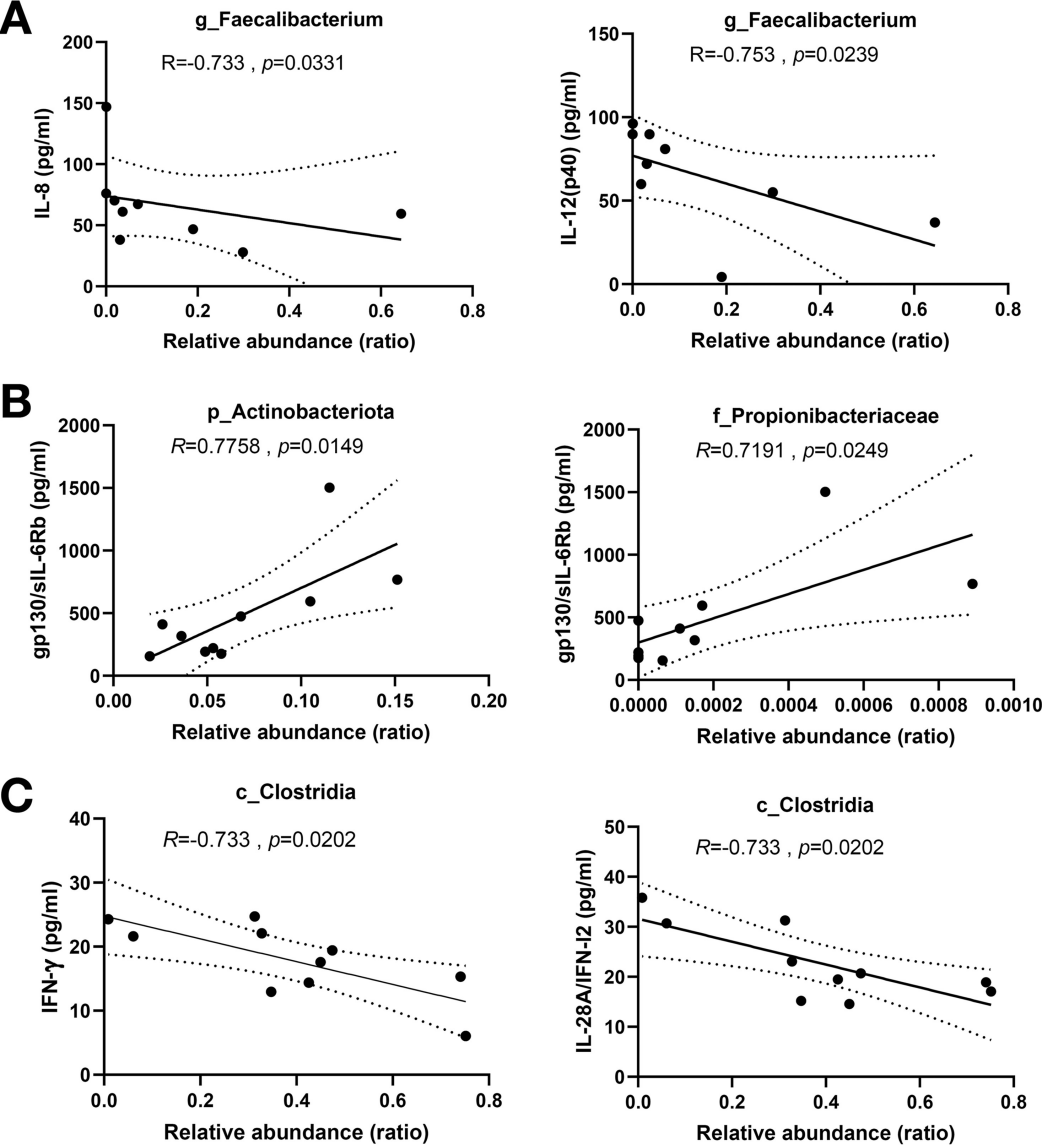
Correlation analysis between increased or decreased intestinal microbiota and plasma concentration of (A) IL-8 and IL-12 (p40), (B) gp130/IL-6Rb, and (C) IFN-γ and IL-28A/IFN-I2 in COVID-19 patients.

Because changes in the gut microbiota may contribute to the immune response, we next performed a correlation analysis between plasma cytokine concentrations and changes in the gut microbiota. The abundance of *Faecalibacterium*, which belongs to class Clostridia, decreased in the patients and was inversely correlated with IL-8 and IL-12 (p40) levels (Fig. 5A). The enrichment of the Actinobacteria phylum and the Propionibacteriaceae family observed in the LEfSe analysis, was positively correlated with gp130/sIL-6Rb level (Fig. 5B). Furthermore, the abundance of the class Clostridia, which was reduced in COVID-19 patients, was inversely correlated with interferon (IFN)-γ and IL-28A/IFN-I2 levels (Fig. 5C).

## DISCUSSION

Cytokines induce inflammation as a host immune response to infection, and as observed in COVID-19, some cases are thought to release excessive or uncontrolled amounts of cytokines, and this cytokine storm can cause fatal organ failure, including the heart, lung, and kidneys, leading to death (16). Thus, the pathophysiology of COVID-19 could be considered as a disease dependent on host-side responses. In this study, we observed changes in the intestinal microbiota of hospitalized patients with COVID-19 over the time course from disease onset to recovery, and we found correlations between microbiota composition and several cytokine and chemokine levels.

In the current study, we hypothesized that the intestinal microbiota changes during the period from disease onset to recovery. We divided stool samples collected from hospitalized patients into groups according to the number of days since the onset of illness. The findings showed that in the first 3 weeks after the onset of illness, four main classes (Clostridia, Bacilli, Coriobacteriia, and Bacteroidia) showed alterations compared with the composition noted in healthy subjects. In particular, the abundance of Firmicutes was observed to decrease immediately after disease onset, which is consistent with the findings of previous reports (9, 17). The gut microbiota observed in this study can be seen as a dysbiotic pattern associated with high intestinal oxygenation. In general, healthy gut microbiota is dominated by anaerobic bacteria, whose metabolism increases the production of short-chain fatty acids. A major short-chain fatty acid is butyric acid, which has been shown to maintain an anaerobic environment in the gut by promoting oxygen consumption by epithelial cells and improving intestinal barrier function (18).

Our study showed depletion of butyrate-producing bacteria, mainly *Faecalibacterium*, belonging to the class Clostridia, after infection, and an enrichment of the genera Escherichia and *Shigella* was observed in COVID-19 patients. These genera belong to the family Enterobacteriaceae and can also grow in aerobic environments. Elevated levels of Enterobacteriaceae have been commonly observed in patients with Crohn's disease (CD) (19). Previous reports showed that inflammatory bowel disease is characterized by the secretion of proinflammatory cytokines (TNF, IFN-γ, IL-6, and IL-12) and anti-inflammatory cytokines (IL-10, TGFβ, and IL-35) in local tissues (20, 21), In particular, CD patients reportedly demonstrate an increased production of IL-12, IL-23, IFN-γ, and IL-17 (20). In the present analysis, increased levels of IL-12 (p40) and IL-10 were observed depending on the severity of the disease (Fig. 4), and the expression levels of IFN-γ and IL-12 (p40) were inversely correlated with the presence of class Clostridia and *Faecalibacterium* (Fig. 5).

Together with the observed enrichment of Escherichia and *Shigella*, our results suggest that the intestinal environment of COVID-19 patients is similar to that of patients with CD and may lead to systemic inflammation via the gastrointestinal tract.

In patients with COVID-19, enrichment of the phylum Actinobacteria, mainly the class Coriobacteriia, was observed immediately after onset. An interesting observation was that enrichment of the genus *Eggerthella*, which belongs to the class Coriobacteriia, was persistently observed in COVID-19 patients from admission to after recovery. This observation is consistent with the findings of a previous report (22). Recent reports have suggested that *Eggerthella* enrichment is associated with rheumatic diseases (23). It has been reported in a mouse model of rheumatic disease that *Eggerthella* regulates Th17 activation through its own metabolites and increases intestinal permeability. The enriched *Eggerthella* in COVID-19 patients may increase intestinal permeability, leading to gastrointestinal symptoms. Considering that leaky gut induces translocation of intestinal bacteria, this could be a trigger for systemic inflammation that leads to severe disease in COVID-19 patients. In view of the above, *Eggerthella* may be a potential therapeutic target for managing COVID-19 in the future.

Determining which factors contribute to the changes in the microbiome composition of patients will help to understand the pathogenesis of COVID-19. In the present analysis, there was no significant difference in the changes in the gut microbiome by the severity of the disease. Presumably, the profile of the gut microbiota in the normal state of the individual before infection may be responsible for the magnitude of the changes in the gut microbiota caused by SARS-CoV-2 infection. Although not analyzed in the present study, the amount of virus shed into the intestinal tract may also directly affect the changes. We also recently found that in acute hepatitis A infection, the virus is shed into the intestinal tract in parallel with the onset of acute hepatitis, resulting in transient dynamic changes in the intestinal microbiota. The mechanism by which these transient changes are involved in the onset of the disease remains unclear.

This study mainly included patients with mild to moderate disease, but the degree of inflammation varied according to the severity. Compared with those in mild disease, levels of inflammatory factors such as IL-2, IL-12 (p40), and IL-10 were increased in moderate disease, and these factors have been previously reported to be increased in patients with COVID-19. In the present study, the increase in Actinobacteria, mainly Propionibacteriaceae, was positively correlated with sIL-6Rb level. sIL-6Rb acts as a trans-signal for IL-6 and its level correlates with the severity of COVID-19 (24). Analysis of nasal microbial profiles collected from COVID-19 positive and negative subjects using nasopharyngeal swabs found a higher relative abundance of Propionibacteriaceae in COVID-19 patients (25). The presence of class Clostridia was negatively correlated with IFN-γ levels; the amount of *Faecalibacterium* was negatively correlated with the levels of proinflammatory cytokines IL-8 and IL-12 (p40). In addition to the previously reported reduction of Firmicutes phylum including the class Clostridia in COVID-19 patients (8, 17), the class Clostridia has been reported to have immunomodulatory effects, suggesting that depletion of these useful bacteria from the early stages of infection may have some effect on activating the immune response against COVID-19. These results suggest a strong link between gut bacteria and inflammation.

One of the limitations of this study is that it mainly analyzed the gut microbiota of mild to moderate cases, and therefore, it did not extend to the analysis of the association between disease severity and gut microbiota. We observed no correlation of patient background, such as gender and age, to alteration of gut microbiota, but this may be due to the limited number of cohorts. In this study, however, changes in the gut microbiota were observed from the early stages of infection, and correlations were found between the composition of the gut microbiota and the levels of several proinflammatory cytokines that have been reported to be associated with COVID-19. Furthermore, changes in the gut environment, including the gut microbiota, were observed in patients, demonstrating the importance of the gut microbiota in understanding the pathogenesis of COVID-19. This finding implies that the gut environment may have an impact on the severity of COVID-19. Although the number of analyses was small, changes in the bacterial flora were observed even after hospital discharge in our study, suggesting that it takes time for the intestinal environment to return to normal. This observation is consistent with the results of a previous study that found that the richness of the gut microbiota had not recovered in COVID-19 patients for 6 months after convalescence (11). This continued dysbiosis may be related to the so-called post-COVID-19 syndrome, in which patients complain of some disorders even after recovery (26). Long-term follow-up of patients with COVID-19 is essential for clarifying the causal relationship between intestinal microbiota and long-term effects of COVID-19. As we come to understand the detailed role of the gut microbiota in disease progression, the signaling pathways of the gut–microbiota peripheral axis will be elucidated, and new concepts of pathogenesis will emerge.

Finally, because the profile of commensal gut microbiota is known to vary among countries, the present results may not be applicable universally. However, our results provide solid evidence of the correlation between gut microbiota and inflammation and propose the importance of gut microbiota and intestinal environment in understanding the pathogenesis of COVID-19. Furthermore, more extensive cross-sectional analyses are needed to understand the role of gut microbiota in SARS-COV-2 infection.

## MATERIALS AND METHODS

### Subject recruitment and sample collection.

Between February and August 2020, we collected stool and blood samples from 22 COVID-19 patients who had been hospitalized at the IMSUT hospital, Institute of Medical Science, University of Tokyo, Japan. Patients with COVID-19 were classified into three severity groups based on a previous report (27). If the patient had various signs and symptoms of COVID-19 (fever, cough, sore throat, etc.) but no dyspnoea or computed tomography (CT) images showing signs of pneumonia, the disease was classified as mild. If CT images showed signs of pneumonia accompanied by fever, respiratory symptoms, and oxygen saturation ≥94%, it was classified as moderate. If CT images showed signs of pneumonia and oxygen saturation <94%, it was classified as severe. Of the 22 patients, 8 were also using antibiotics at the time of admission. We collected blood and stool samples from the patients during hospitalization; six discharged patients provided stool samples on the follow-up day (1–2 weeks after discharge). Stool specimens were collected and provided by the patients themselves in the hospital. Stool specimens after discharge from the hospital (>21 days from onset) were brought to the hospital by the patient under refrigeration. The blood and stool specimens were immediately transported to a laboratory located near the hospital. We stored the stool samples at −80°C before DNA preparation, whereas blood samples were separated into peripheral blood mononuclear cells and plasma fractions using density gradient centrifugation. The plasma fraction was stored at −80°C.

As a control group, historical cohort data of 40 healthy individuals who were recruited in a previous study (13) were used. Those who consumed antibiotics within the previous 2 weeks were excluded.

### Ethical approval.

This study was approved by the Institutional Review Board of the Institute of Medical Science, University of Tokyo (IMSUT; approval number reference 28-55-0330, 2019-71-0201). We confirmed that all methods were performed in accordance with the relevant guidelines and regulations. This study was conducted in accordance with the Declaration of Helsinki and written informed consent for sample collection and subsequent analysis was provided by all participants (healthy individuals and patients) before enrollment.

### Preparation of bacterial fractions from fecal samples.

We prepared bacterial pellets from frozen fecal samples. Briefly, stool (1 g) was added to 10 mL of SM-plus buffer (100 mM NaCl, 50 mM Tris-HCl [pH 7.4], 8 mM MgSO_4_·7H_2_O, 5 mM CaCl_2_·2H_2_O, and 0.01% [wt/vol] gelatin). Bacterial suspensions were then filtered through a 100 μm cell strainer (Corning, Inc., Corning, NY, USA). The filtered bacterial suspensions were used for DNA extraction.

### DNA extraction, amplification, and 16S rRNA gene sequencing.

We extracted DNA from the fecal sample-derived bacterial fraction, as previously described (13). The 16S rRNA gene libraries were prepared according to the 16S Metagenomics Sequencing Library Preparation guide (Part #15044223 Rev. B; Illumina, San Diego, CA, USA). Briefly, the hypervariable V3–V4 region of the 16S rRNA gene was amplified using specific primers: forward (5′-ACACGACGCTCTTCCGATCTCCTACGGGNGGCWGCAG-3′) and reverse (5′-GACGTGTGCTCTTCCGATCTGACTACHVGGGTATCTAATCC-3′), comprising Illumina adapter overhang nucleotide sequences (underlined) (28). Next, adapter ligation for PCR amplicons was performed using NEBNext Multiplex Oligos for Illumina (Dual Index Primers Set 1; New England Biolabs, Ipswich, MA, USA). Sequencing was performed on the Illumina MiSeq system (Illumina), using the MiSeq reagent kit v3 (600-cycle) with a 20% PhiX (Illumina) spike-in.

### Sequencing and statistical analyses.

Sequences were quality filtered, denoised, and analyzed using Quantitative Insights Into Microbial Ecology 2 (QIIME 2 version 2019.4) as previously reported (29). In brief, DADA2 was used to denoise the paired-end reads into amplicon sequence variants (30). A bacterial taxonomic classification was assigned to the resulting amplicon sequence variants against the SILVA database (release 132) (31). This was trimmed to the V3–V4 region of the 16S rRNA gene using a naive Bayesian classification method (32). The Kruskal-Wallis test was used for statistical analysis of alpha diversity (Shannon index) and analyzed with QIIME2 software (cutoff *P* value: less than 0.05). Operational taxonomic unit (OTU) tables were aligned to an equal sampling depth of 10,000 per sample, sufficient for analysis, by alpha-rarefaction analysis to avoid bias caused by differences in sequence depth. Data were preprocessed as described in ANCOM-II to remove low-abundance or rare taxa before differential presence ratio analysis (33). Metagenomic profile statistical analysis was performed using STAMP version 2.0 (34), and the linear discriminant analysis effect size (LEfSe) method was used to identify differentially abundant taxa (35).

### Plasma cytokine analysis.

The plasma cytokine levels were quantified using the Bio-Plex System (Bio-Rad Laboratories) with the multiplex assay kits, namely, Bio-Plex Pro Human Chemokine Panel (40-Plex #171AK99MR2) and Bio-Plex Pro Human Inflammation Panel 1 (37-Plex #171AL001M) in accordance with the manufacturer’s specifications. To align the time from disease onset, inflammatory marker levels were quantified in plasma samples collected within 7–14 days of disease onset. Pearson’s correlation analysis was conducted to identify relationships between fecal microbiome abundance and cytokine expression.

### Data availability.

Data described in this study are openly available in the DNA Data Bank of Japan (DDBJ) (https://ddbj.nig.ac.jp/DRASearch; accession numbers: DRA012374 and DRA013054).
