# Supplementary material for: Correlation Analysis between Gut Microbiota Alterations and the Cytokine Response in Patients with Coronavirus Disease during Hospitalization
Source: Microbiol Spectr. 2022 Mar 7;10(2):e01689-21. doi: 10.1128/spectrum.01689-21 (PMC9045125; doi:10.1128/spectrum.01689-21)
Supplement: SUPPLEMENTAL FILE 1 — Supplemental material. Download SPECTRUM01689-21_Supp_1_seq8.pdf, PDF file, 1.1 MB [file spectrum01689-21_supp_1_seq8.pdf]

# Supplemental Fig.1

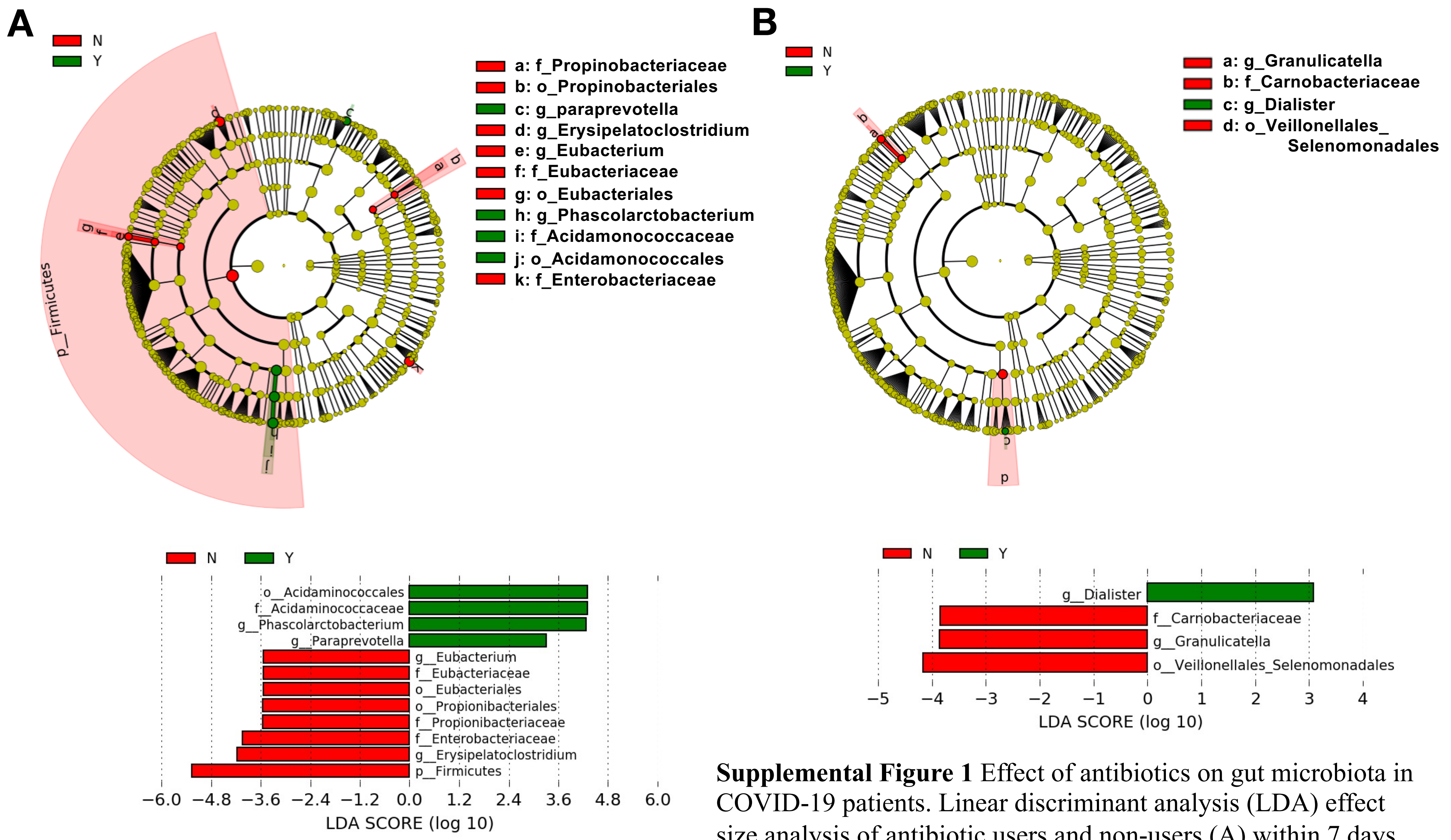


**Supplemental Figure 1** Effect of antibiotics on gut microbiota in COVID-19 patients. Linear discriminant analysis (LDA) effect size analysis of antibiotic users and non-users (A) within 7 days and (B) 8–14 days from onset (LDA >3). Y; antibiotic users, N; antibiotic non-users
